# Moderate calcium stress improves antioxidant phytochemical quality of *Phedimus aizoon* L. through flavonoid and glutathione dual ROS–Ca²^+^ detoxification strategy

**DOI:** 10.3389/fpls.2026.1737412

**Published:** 2026-04-29

**Authors:** Ke Zhang, Chong Luo

**Affiliations:** 1 School of Life Sciences, Guizhou Normal University, Guiyang, China; 2 College of Teacher Education, Guizhou Normal University, Guiyang, China

**Keywords:** adaptive trade-off, biphasic response, calcium stress, flavonoid–glutathione coordination, ionic homeostasis, multi- omics, *Phedimus aizoon*, ROS detoxification

## Abstract

**Background:**

Elevated soil calcium in karst regions significantly influences plant development and phytochemical composition. This study aimed to elucidate the physiological and molecular basis of high-calcium adaptation in P. aizoon and to evaluate the effects of exogenous calcium on its edible and medicinal quality.

**Methods:**

*P. aizoon* seedlings were cultivated under five CaCl_2_ concentration treatments. Growth responses, assessed by plant height and crown width, were used to identify three representative treatment groups for further analyses. Edible tissues from these plants were subsequently subjected to nutritional assessment, stress-related physiological measurements, antioxidant and hypoglycaemic activity assays, and metabolic and transcriptional profiling.

**Results:**

*P. aizoon* exhibited a clear biphasic response to calcium gradients. Moderate calcium (30 mM) optimized growth performance and significantly enhanced the accumulation of total flavonoids (1.51-fold), polyphenols, and glutathione (GSH), together with elevated activities of antioxidant enzymes (SOD, CAT, GST, and APX). In contrast, severe calcium stress (150 mM) suppressed biomass accumulation while maintaining high antioxidant capacity. Multi-omics analyses indicated that calcium-responsive changes in flavonoid and glutathione metabolism were associated with the induction of stress-responsive transcription factors, including members of the MYB and WRKY families.

**Conclusion:**

These findings suggest that *P. aizoon* adjusts growth and antioxidant metabolism across a calcium gradient through coordinated transcriptional and metabolic reprogramming. Enhanced flavonoid and glutathione metabolism may contribute to redox homeostasis under elevated calcium conditions. This study provides a conceptual framework for understanding plant responses to high-calcium environments.

## 1 Introduction

*Phedimus aizoon* is an edible and medicinal plant widely distributed in China, Russia, Mongolia, Japan, North Korea, and Mexico. It contains many bioactive compounds including flavonoids, polyphenols, phytosterols, and alkaloids, and shows multiple pharmacological activities including antioxidant, anti-fatigue, antibacterial, anticancer, hemostatic, sedative, and hypnotic activities (Wang et al., 2024). In addition to its nutritional and medicinal value, *P. aizoon* has a wide range of physiological adaptability to different environmental stresses. *P. aizoon* exhibits strong cold resistance, which can be enhanced through symbiosis with arbuscular mycorrhizal fungi (AMF) or treatment with exogenous salicylic acid (Chen et al., 2019). Similarly, *P. aizoon* L. tolerates saline soil with 100 mmol L^-^¹ NaCl, contributing to a reduction in soil salinity. Moreover, salt stress leads to elevated levels of tannins and flavonoids in the plant (Hu et al., 2021; Jin and Wang, 2023; Zhang et al., 2020; Wang et al., 2010). Moreover, *P. aizoon* shows strong phytoremediation ability by accumulating Cd and Pb in its tissues. This accumulation is influenced by Zn²^+^ and can be further enhanced through symbiosis with arbuscular mycorrhizal fungi (AMF) (Chen et al., 2019). Soil acidity strongly affects the physiological activities of *P. aizoon* Mild acidification (pH 5.6) enhances root growth and activity, whereas severe acidification (pH 3.4) causes chloroplast damage and inhibits photosynthesis. Under moderately acidic conditions (pH 6.0), the greatest Pb tolerance occurs at a Pb²^+^ concentration of 20 mg L^-^¹ (Chen et al., 2019). Due to its strong tolerance to drought, cold, and poor soils, *P. aizoon* is widely used for windbreaks, sand fixation, and urban greening (Wang et al., 2019).

*P. aizoon* is recognized as a “high-calcium vegetable”, with calcium levels reaching up to 0.315% in its tender leaves (Yi, 2000). However, it is seldom discovered in naturally calcium-rich areas like the southern karst areas of China. In fact, how the plant with naturally high tissue Ca²^+^ content responds to excess external calcium and whether it can adapt to high calcium environment remain to be further explored. Additionally, how would elevated Ca²^+^ affect its nutritional and medicinal properties including antioxidant and hypoglycaemic activities? The answers to these questions would broaden our understanding of plant mineral-stress biology and facilitate the breeding of calcium-tolerant high-quality cultivars.

Calcium is essential for plant growth, development and stress responses. Calcium (Ca²^+^) serves as a universal second messenger, playing a pivotal role in plant growth, development, and the mediation of responses to various environmental stimuli (Negi et al., 2023). Recent studies have highlighted that the calcium-signaling connection is fundamental to the intricate regulatory networks governing plant resilience under stress (Narwal et al., 2024a). Many previous studies have characterized the regulatory networks of Ca²^+^ signaling and calcium homeostasis in plants. For example, Cold-responsive Ca²^+^-ATPases were localized in guard cells and involved in cold responses (Schiøtt and Palmgren, 2005). Overexpression of OsACA6 improved plant tolerance to salt and drought stress (Huda et al., 2013). In addition, Ca²^+^-dependent protein kinase CPK3 was involved in salt acclimation in *Arabidopsis* (Mehlmer et al., 2010). Ca²^+^/calmodulin was reported to promote root growth under osmotic stress (Niu et al., 2017). Moreover, calcium supplementation improved heavy metal stress tolerance in crops (Kanu et al., 2019; Wang et al., 2021). Recent findings have laid a sound basis for studying Ca²^+^ homeostasis and signaling in plants. However, most research so far has dealt with calcium deficiency or moderate stress, often using model species. Studies on plants that naturally accumulate high levels of calcium are still limited, and how these plants adapt to high-calcium environments remains unclear.

As a typical calcium-accumulating species, *P. aizoon* provides a useful system for examining plant responses to elevated external calcium. However, how calcium-accumulating plants respond to high-calcium conditions, and how such responses are associated with edible and medicinal quality traits, remains insufficiently understood. In this study, we exposed it to different CaCl_2_ concentrations and evaluated growth performance, key physiological traits, amino acid composition, antioxidant capacity, hypoglycaemic activity, and metabolic and transcriptomic changes in the edible shoot tissues. By integrating these datasets, we aimed to characterize the response of *P. aizoon* to CaCl_2_-based calcium treatment under controlled soilless conditions, identify treatment levels associated with growth inhibition or tolerance, and assess potential changes in nutritional and medicinal quality. The findings provide a basis for understanding the response of the plant to soluble calcium treatment, while also offering preliminary information relevant to its cultivation and quality management under calcium-rich growing conditions.

## 2 Materials and methods

### 2.1 Plant materials

*P aizoon* seedlings were provided by the wild vegetable base in Jiuzhou Town, Huangping County, Guizhou Province, China. The species was identified by Professor Luo Chong from the School of Teacher Education, Guizhou Normal University, based on morphological characteristics.

### 2.2 Plant culture

Uniform seedlings were selected and transplanted into plastic pots (20 cm in diameter) filled with expanded vermiculite, leaving approximately 1 cm below the pot rim. Expanded vermiculite was used as a soilless growth substrate because it contains negligible background calcium relative to the Ca²^+^ supplied via nutrient solution and therefore allows better control of external calcium treatment (Papadopoulos et al., 2008; Wu Y. et al., 2023). All pots were maintained in a greenhouse under controlled conditions (25 °C, 75% relative humidity) and irrigated regularly with Hoagland nutrient solution.

### 2.3 Screening of CaCl_2_ concentration

In this study, CaCl_2_ was selected as the exogenous calcium source to simulate calcium stress and evaluate flavonoid biosynthesis strategies, avoiding the potential confounding nutritional effects of nitrate enrichment associated with Ca(NO_3_)_2_ (Liu et al., 2025). This design aligns with recent protocols for investigating calcium-mediated secondary metabolism in horticultural species (Narwal et al., 2024a). To determine the optimal concentration range for *P. aizoon* under these conditions, a preliminary gradient (0, 5, 30, 100, and 150 mM) was applied based on established stress models in other medicinal plants (Sabir et al., 2012). At the early seedling stage (approximately 10 days after planting), plants were treated with CaCl_2_ solutions on days 10, 20, and 30 after planting, with 0 mM serving as the control.

At 40 days after planting, plant height and crown width were measured to assess morphological responses. Based on these preliminary screening results (see Section 3.1), three representative concentrations (0, 30, and 150 mM CaCl_2_) were selected for subsequent physiological, biochemical, metabolomic, and transcriptomic analyses. To ensure that these analyses reflected the nutritional and medicinal quality of the consumed plant tissues, edible portions corresponding to common commercial harvest practice were collected. Specifically, the upper 5 cm of tender stems and leaves were harvested, immediately frozen in liquid nitrogen, and stored at −80 °C for further analysis. Each sample weighed approximately 2.0 g.

### 2.4 Determination of amino acid content

Based on the screening results of plant height and crown width (Section 2.3), edible parts of *P. aizoon* treated with 0 mM, 30 mM, and 150 mM CaCl_2_ were collected. Amino acid contents were determined according to previously reported methods (do Nascimento et al., 2020; Lu et al., 2020).

### 2.5 Determination of total calcium and soluble calcium contents

Edible parts of *P. aizoon* grown under the selected CaCl_2_ treatments (0 mM, 30 mM, and 150 mM) were harvested after plant height and crown width measurements. Total and soluble calcium contents were measured as previously described (Wu Q. et al., 2023).

### 2.6 Measurement of physiological and biochemical indicators

Edible tissues of *P. aizoon* subjected to the selected CaCl_2_ treatments (0 mM, 30 mM, and 150 mM) were collected after plant height and crown width measurements. The contents of malondialdehyde (MDA), soluble sugar, soluble total protein (STP), proline, total flavonoids, total polyphenols, and L−ascorbic acid (ASA), as well as the activities of superoxide dismutase (SOD), peroxidase (POD), catalase (CAT), ascorbate peroxidase (APX), and glutathione S−transferase (GST), were determined following the manufacturer’s instructions using Grace assay kits (Suzhou Grace Biotechnology Co., Ltd., Suzhou, China).

### 2.7 Antioxidant and in vitro hypoglycaemic activity

Following plant height and crown width assessment, edible tissues of *P. aizoon* under the selected CaCl_2_ treatments (0 mM, 30 mM, and 150 mM) were used to evaluate antioxidant capacity (DPPH, ABTS, and total antioxidant capacity using the FRAP method) and in vitro hypoglycaemic activities (α−amylase and α−glucosidase inhibition) using Grace assay kits (Suzhou Grace Biotechnology Co., Ltd., Suzhou, China) according to the manufacturer’s instructions.

### 2.8 Sample preparation and metabolite extraction

Harvesting of the edible portions of *P. aizoon* exposed to 0 mM, 30 mM, and 150 mM CaCl_2_ was carried out according to plant height and crown width data. Samples were thawed at 4 °C, extracted with precooled methanol:acetonitrile:water (2:2:1, v/v), processed by vortexing, low-temperature ultrasonication (30 min), and centrifugation (14,000 g, 4 °C, 20 min). Supernatants were vacuum-dried, redissolved in acetonitrile:water (1:1, v/v), and centrifuged again.

The redissolved supernatants were analyzed using an Ultra-High Performance Liquid Chromatography (UHPLC) system (Thermo Fisher Scientific) coupled with a Q-Exactive mass spectrometer (Thermo Fisher Scientific). Metabolite separation was performed on a Waters ACQUITY UPLC BEH C18 column (2.1 mm × 100 mm, 1.7 μm) maintained at 40 °C. The mobile phase consisted of 0.1% formic acid in water (A) and acetonitrile (B), with a flow rate of 0.3 mL/min. Mass spectrometry was conducted in both positive and negative electrospray ionization (ESI) modes.

Raw data were processed using the XCMS package in R for peak picking, alignment, and quantification. To ensure comparability across samples, total ion chromatogram (TIC) normalization was applied to the peak area data. Metabolite annotation was performed by matching sample m/z data and MS/MS fragments against the KEGG database (http://www.kegg.jp/kegg/) and an in-house spectral library. According to the Metabolomics Standards Initiative (MSI) guidelines, the metabolite identification confidence in this study reached Level 2 (putatively identified compounds based on spectral library matching).

PLS-DA was used to maximize metabolic separation. OPLS-DA variable importance in projection (VIP) analysis was applied; metabolites with FC > 1.5 or FC < 0.67 and p < 0.05 were considered differential.

### 2.9 RNA-seq and qRT-PCR

Edible components of *P. aizoon* plants treated with 0 mM, 30 mM, and 150 mM CaCl_2_ were thawed at 4 °C and sampled (three biological replicates per treatment, nine total). RNA extraction and library preparation were performed by Shanghai Origingene Co., Ltd. Total RNA quality was assessed by agarose gel electrophoresis, NanoPhotometer (Implen, Germany), and Agilent 2100 Bioanalyzer (Agilent Technologies). Poly(A) mRNA was purified with oligo(dT) magnetic beads, rRNA removed, and mRNA fragmented for cDNA synthesis, double−stranded cDNA production, and library construction. Libraries were end−repaired, adapter−ligated, PCR−amplified, quantified, diluted, and size−assessed; those > 2 nM effective concentration were sequenced (Illumina platform). Clean reads were analyzed, and DEGs (FDR < 0.05, |log_2_FC| > 1) identified using DESeq2 (Love et al., 2014). The qPCR was used to verify transcript levels.

The raw sequencing data supporting this study have been deposited in the Genome Sequence Archive (Li et al., 2025) at the National Genomics Data Center (Zhao et al., 2025), China National Center for Bioinformation/Beijing Institute of Genomics, Chinese Academy of Sciences, under accession number CRA032169 (https://ngdc.cncb.ac.cn/gsa).

### 2.10 Data analysis

Genes related to metabolites were identified via the Phytozome database. Venn diagrams, correlation heatmaps, and expression heatmaps were generated with TBtools (Chen et al., 2020). Data organization: Microsoft Excel (Office 2016); graphing: GraphPad Prism 10; statistics: SPSS 25.0, one−way ANOVA (Chen et al., 2023) with ≥ 3 biological replicates for all experiments.

## 3 Results

### 3.1 Effects of Ca²^+^ treatments on plant morphology

**Figure 1:**
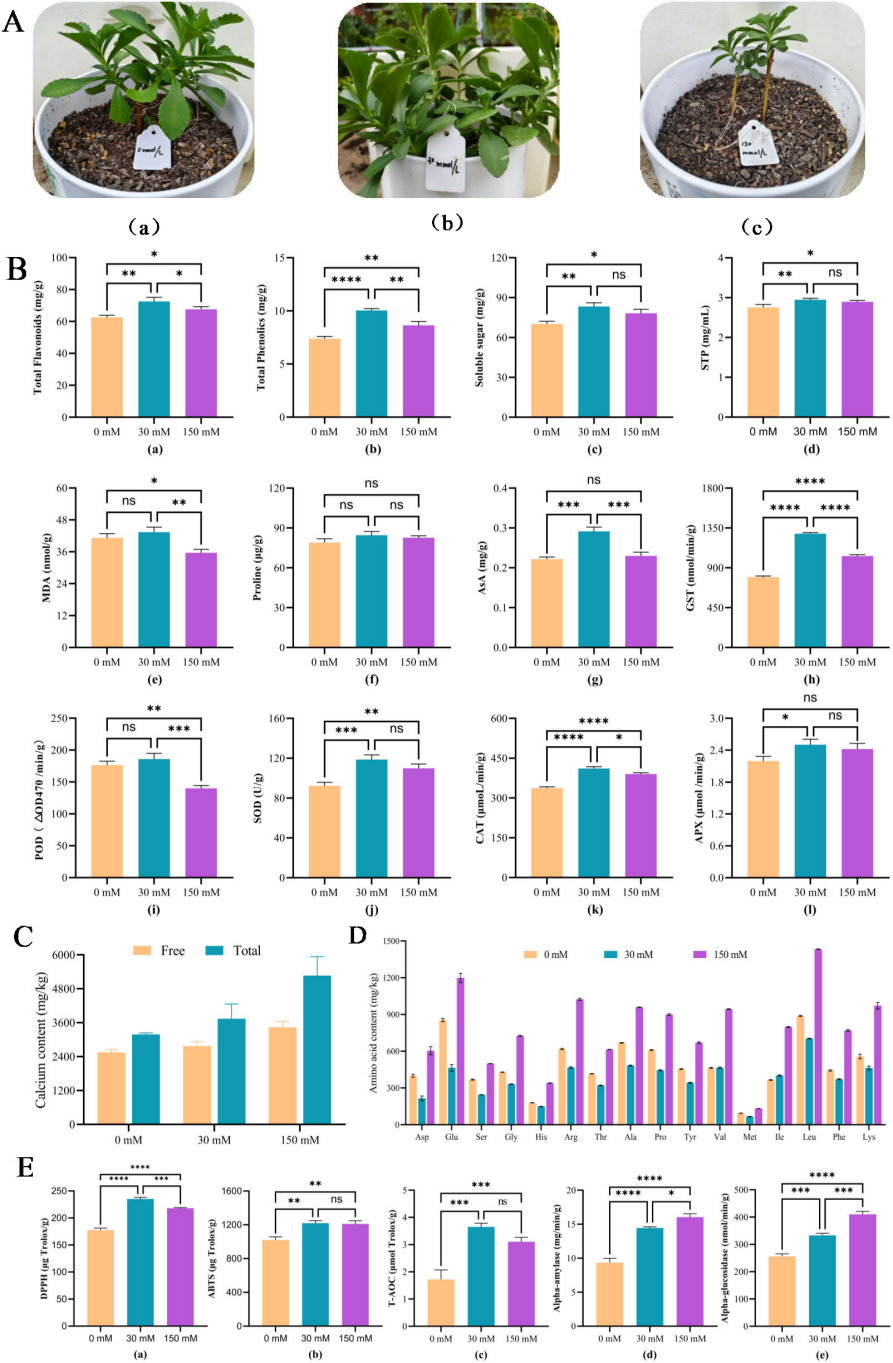
Effects of different Ca²^+^ treatments on morphology and related parameters in *P. aizoon*. **(A)** Representative phenotypes under 0, 30, and 150 mM Ca²^+^. **(B)** Total flavonoids (a), total phenolics (b), soluble sugars (c), soluble total protein (STP) (d), MDA (e), proline (f), ABA (g), GSH (h), POD (i), SOD (j), CAT (k), and APX (l), **(C)** Free and total calcium content. **(D)** Amino acid content. **(E)** DPPH (a), ABTS (b), T-AOC (c), α-amylase, (d), and α-glucosidase (e), Data are means ± SD (n = 3). Asterisks denote significant differences: *p < 0.05, **p < 0.01, ***p < 0.001, and ****p < 0.0001 (One-way ANOVA with Tukey’s test); ns, not significant.

Plant growth exhibited a clear response to Ca²^+^ availability, as illustrated in **Figure 1A** and **Table 1**., *P. aizoon* growth exhibited a concentration-dependent biphasic response to CaCl_2_. Both plant height and crown breadth peaked at 30 mM (18.50 ± 0.50 cm and 22.67 ± 0.58 cm, respectively), significantly exceeding the control. Conversely, these parameters declined to 14.53 ± 0.06 cm and 19.17 ± 0.76 cm at 150 mM, reflecting marked growth inhibition under high calcium stress In contrast, exposure to 150 mM Ca²^+^ resulted in reduced growth performance, characterized by lower plant height (14.6 cm) and crown breadth (18.6 cm) than those observed in the control. These findings indicate a concentration-dependent growth response, in which moderate Ca²^+^ supply favors morphological development, whereas excessive Ca²^+^ constrains plant growth.

**Table 1:** Effects of different CaCl_2_ concentrations on the morphological traits of *P. aizoon*.

CaCl_2_ concentration (mM)	0	5	30	100	150
Plant Height (cm)	14.17 ± 0.29	15.50 ± 0.50	18.50 ± 0.50	16.17 ± 0.76	14.53 ± 0.06
Crown Breadth (cm)	19.00 ± 0.00	18.83 ± 0.29	22.67 ± 0.58	21.00 ± 1.00	19.17 ± 0.76

### 3.2 Ca²^+^-induced alterations in stress-related physiological and biochemical traits

Consistent with the morphological responses, Ca²^+^ supply substantially altered stress-related physiological and biochemical parameters (**Figure 1B**). At 30 mM Ca²^+^, the accumulation of protective secondary metabolites was enhanced, with total flavonoid content increasing from 62.5 to 72.6 mg/g FW and total phenolic content from 7.38 to 10.04 mg/g FW (**Figures 1B-a, b**).

Osmoregulatory capacity also improved under moderate Ca²^+^ supply. Soluble sugar content increased from 70.2 to 83.4 mg/g FW, accompanied by a rise in soluble total protein from 2.75 to 2.95 mg/mL FW (**Figures 1B-c,d**).

Antioxidant defense responses were particularly sensitive to Ca²^+^. At 30 mM Ca²^+^, SOD and CAT activities increased by approximately 28% and 22%, respectively, while GST activity increased by about 1.6-fold relative to the control (**Figures 1B-h, j, k**). By contrast, MDA content declined under 150 mM Ca²^+^, decreasing from 41.24 to 35.64 nmol/g FW (**Figures 1B-e**), suggesting reduced lipid peroxidation under high Ca²^+^ conditions.

### 3.3 Calcium accumulation and distribution under different Ca²^+^ treatments

As shown in **Figure 1C**, increasing Ca²^+^ supply led to a progressive increase in calcium accumulation in *P. aizoon*. Total Ca content increased from 3.18 × 10³ mg/kg FW in the control to 3.74 × 10³ mg/kg FW at 30 mM Ca²^+^, and further reached 5.23 × 10³ mg/kg FW at 150 mM Ca²^+^. A similar trend was observed for soluble Ca²^+^, which increased from 2.55 × 10³ mg/kg FW in the control to 2.77 × 10³ mg/kg FW at 30 mM, and 3.42 × 10³ mg/kg FW at 150 mM Ca²^+^. These results demonstrate a strong capacity of *P. aizoon* to absorb and accumulate Ca under Ca-rich conditions.

### 3.4 Ca²^+^ -dependent modulation of amino acid composition

Ca²^+^ treatments significantly influenced amino acid accumulation patterns (**Figure 1D**). While 30 mM Ca²^+^ was associated with relatively stable amino acid levels, 150 mM Ca²^+^ resulted in pronounced enrichment of multiple amino acids. It is worth noting that glutamate content increased from 854.3 to 1218.1 mg/kg FW, and arginine increased from 618.5 to 1027.3 mg/kg FW under 150 mM Ca²^+^. Several essential amino acids, including leucine, isoleucine, and lysine, showed similar enhancement trends, with leucine reaching 1435.5 mg/kg FW, compared with 888.2 mg/kg FW in the control.

### 3.5 Ca²^+^ -mediated enhancement of antioxidant and hypoglycemic activities

In agreement with the physiological and compositional changes, Ca²^+^ supply significantly affected antioxidant capacity and bioactive properties (**Figure 1E**). At 30 mM Ca²^+^, ABTS and DPPH radical scavenging activities increased by approximately 19% and 33%, respectively, relative to the control (**Figures 1E-a, b**). In parallel, total antioxidant capacity more than doubled, increasing from 1.72 to 3.65 μmol Trolox/g FW (**Figures 1E-c**).

Enzyme inhibitory activities followed a comparable pattern. α-Amylase activity increased from 9.35 to 16.05 mg/min/g FW, while α-glucosidase activity increased from 256.2 to 410.2 nmol/min/g FW at 30 mM Ca²^+^ (**Figures 1E-d, e**). Although slightly lower values were observed at 150 mM Ca²^+^, they remained substantially higher than those of the control.

Overall, these results indicated that flavonoid, phenolic, and soluble sugar contents were accumulated, and the antioxidant defense system, including SOD, CAT, GST, and APX, was activated by 30 mM calcium stress, which enhanced the stress tolerance of *P. aizoon.* In contrast, high calcium stress (150 mM) diminishes these promoting effects, leading to variable physiological responses and growth inhibition.

### 3.6 Global response characteristics of differentially expressed genes under calcium stress

**Figure 2:**
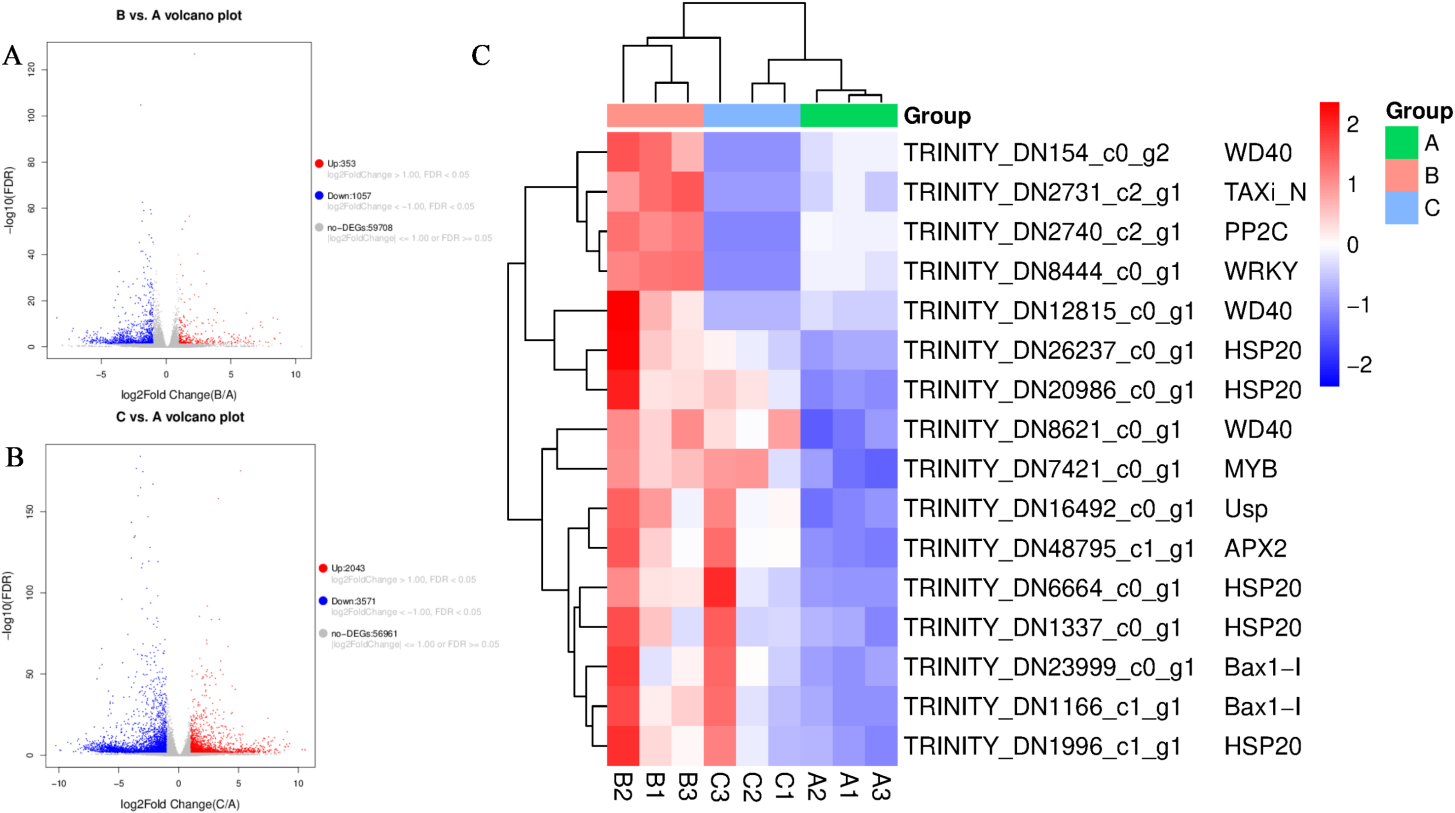
Statistics and expression patterns of differentially expressed genes (DEGs) under calcium stress. **(A, B)** Volcano plots showing DEGs in the **(A)** 30 mM and **(B)** 150 mM CaCl_2_ treatments compared to the control. **(C)** Heatmap of nine representative stress-resistance genes selected from common upregulated DEGs across treatments.

Differentially expressed gene (DEG) analysis revealed significant transcriptional reprogramming under calcium stress. Under moderate stress (30 mM CaCl_2_), a total of 1,410 DEGs were identified, of which 353 (25.0%) were upregulated and 1,057 (75.0%) were downregulated (**Figure 2A**). In contrast, severe stress (150 mM CaCl_2_) induced a much broader transcriptional shift, with 5,614 DEGs detected. Among these, 2,043 genes (36.4%) were upregulated and 3,571 (63.6%) were downregulated (**Figure 2B**). The proportional increase in upregulated genes under severe stress suggests intensified transcriptional activation of defense−related processes, although overall suppression of growth−related pathways became more pronounced.

When classified into functional categories, upregulated genes were mainly enriched in antioxidant defense and ion transport clusters (62%), while downregulated genes were mainly enriched in photosynthesis and cell elongation clusters (58%). This is consistent with the upregulation of defense functions and downregulation of growth and energy metabolism in *P. aizoon* upon calcium stress. Taken together, these results suggest that *P. aizoon* adopts a transcriptional strategy of “prioritizing defense over growth” to respond to elevated calcium stress.

To validate key candidate genes, hierarchical clustering analysis was performed (**Figure 2C**). Nine stress−associated genes were consistently upregulated across treatments, including transcriptional regulators and stress−response proteins such as *WD40*, *TAXi_N*, *PP2C*, *WRKY*, *MYB*, *HSP20*, *APX2*, *Bax1−I*, and *Usp*. These genes are functionally linked to flavonoid metabolism, antioxidant activity, ion homeostasis, and general stress resistance, supporting their critical roles in mediating adaptive responses to calcium stress.

### 3.7 GO functional enrichment characteristics of differentially expressed genes

**Figure 3:**
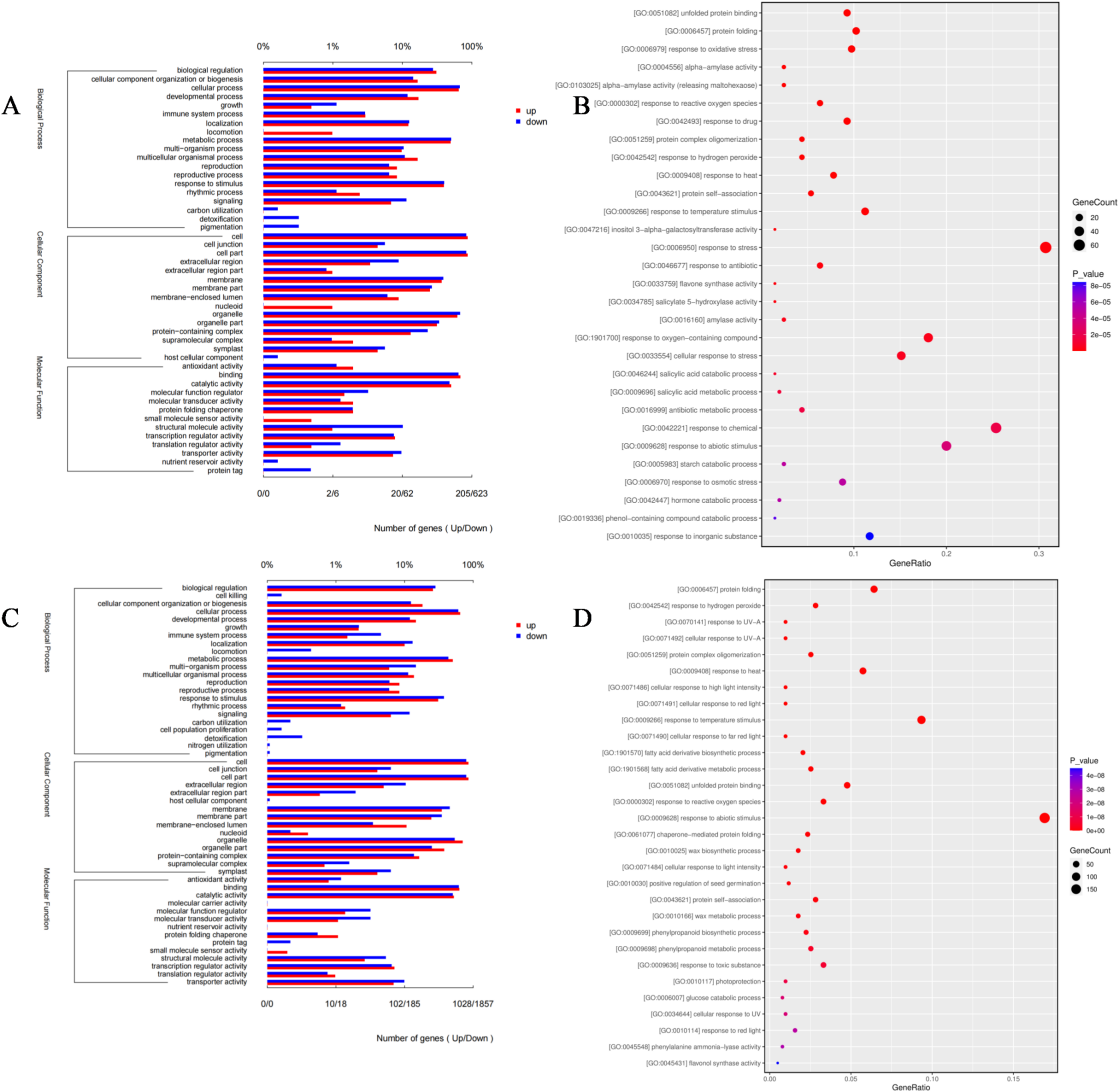
Gene Ontology (GO) functional enrichment analysis of differentially expressed genes (DEGs) under calcium stress. **(A, B)** GO enrichment categories of DEGs in the 30 mM CaCl_2_ treatment (Group B). **(C, D)** GO enrichment categories of DEGs in the 150 mM CaCl_2_ treatment (Group C).

Gene Ontology (GO) enrichment analysis revealed distinct transcriptional reprogramming at moderate (30 mM) and severe (150 mM) calcium stress levels. At 30 mM CaCl_2_, the biological process (BP) category “response to stimulus” contained 667 upregulated genes, among which the subcategory “response to oxidative stress” accounted for 32% (213 genes) (**Figures 3A, B**). This cluster also contained several well-known genes involved in antioxidant signaling during calcium stress, such as *APX2* and *HSP20*, which are directly involved in ROS detoxification. In the molecular function (MF) category, “antioxidant activity” contained 108 upregulated genes, which accounted for ~10% of all MF-enriched genes. It is worth noting that the *glutathione reductase* gene (*GR*), which plays an important regulatory role in GSH regeneration, was upregulated. For cellular component (CC), 52 membrane genes were upregulated, including one calcium ion transporter (*TRINITY_DN24305_c0_g2_i4*), whose expression was negatively correlated with cytoplasmic calcium concentration (r = –0.76), suggesting its potential role in ion homeostasis.

Compared with 30 mM, severe stress (150 mM) induced broader enrichment (**Figures 3C, D**). The number of upregulated “detoxification” genes increased from 54 to 185 (a 3.4-fold increase), while the proportion of “cellular component organization” genes increased from 8% to 12%. Endoplasmic reticulum repair-related genes, such as *HSP20*, were further upregulated, reflecting enhanced requirements for structural repair under high calcium conditions. Moreover, enrichment in “transporter activity” increased by 40%, with ABC transporter family genes comprising 65% of this category. These transporters are likely involved in vacuolar partitioning of secondary metabolites, consistent with metabolomic observations of flavonoid accumulation within vacuoles.

### 3.8 KEGG pathway enrichment and core metabolic pathway map of differentially expressed genes

**Figure 4:**
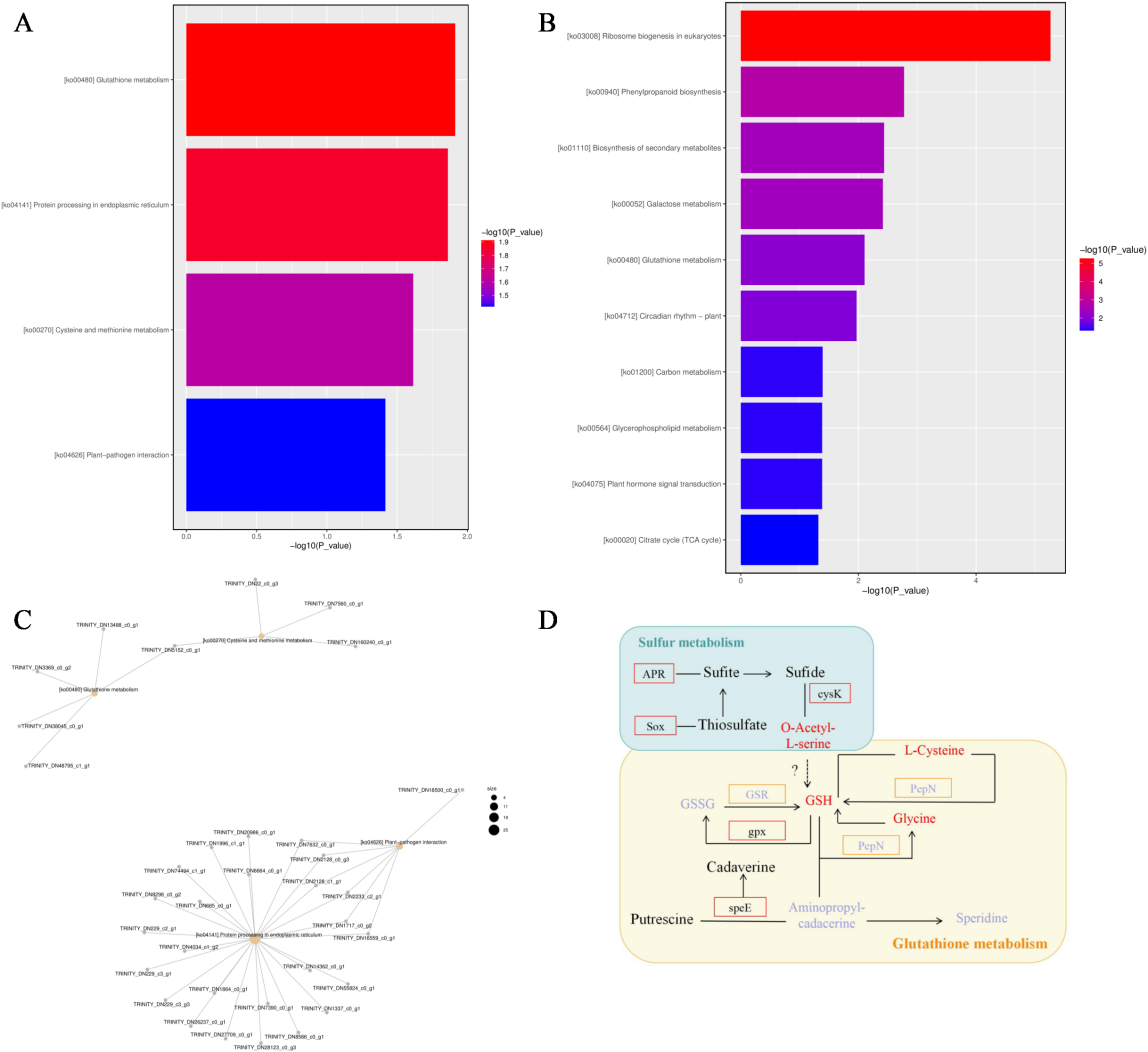
**(A, B)** Top enriched KEGG pathways of DEGs under 30mM and 50mM Ca stress treatment. The rectangle length represent gene count, and the color represent P value. The top 4 pathways are shown, ranked by P value. **(C)** Joint analysis of metabolites and genes in the top four enriched pathways under 30 mM calcium stress. **(D)** KEGG map of glutathione pathway and Sulfur metabolism pathway under Ca stress. Purple indicates upregulation of moderate stress (30 mM) enzymes and downstream products, and red indicates upregulation of severe stress (150 mM) enzymes and downstream products.

KEGG pathway analysis revealed significant enrichment of stress−responsive pathways under both moderate (30 mM) and severe (150 mM) calcium stress. At 30 mM CaCl_2_, four pathways were significantly enriched: glutathione metabolism (ko00480, –*log*_10_(p-value) = 1.9), protein processing in the endoplasmic reticulum (ko04141), cysteine and methionine metabolism (ko00270), and plant−pathogen interaction (ko04626) (**Figure 4A**). It is worth noting that cysteine metabolism provides precursors for glutathione synthesis; *O−acetylserine sulfhydrylase* expression showed a strong positive correlation with *glutathione synthase* (*GSHS*) (r = 0.87), forming a “substrate supply–product synthesis” coupling network. Within glutathione metabolism, the *γ−glutamyl transferase* gene (*GGT*; *TRINITY_DN3369_c0_g2*) was upregulated, catalyzing the condensation of L−glutamic acid and cysteine into γ−glutamyl−cysteine, a key precursor of glutathione. These results identify glutathione metabolism as the most enriched and central pathway under moderate stress.

Under severe stress (150 mM CaCl_2_), ten significantly enriched pathways were detected (**Figure 4B**), dominated by ribosome biogenesis in eukaryotes (ko03008), phenylpropanoid biosynthesis (ko00940), and glutathione metabolism (ko00480). In this condition, *glutathione peroxidase* (*GPX*) and *spermidine synthase* were upregulated, promoting the conversion of GSH to GSSG through *GPX*−mediated activity, which scavenges H_2_O_2_ and maintains cellular redox balance. The accumulation of GSSG, spermidine, and polyamine derivatives enhanced the cellular antioxidant capacity. Spermidine stabilizes membranes, regulates oxidative homeostasis, and augments the activity of antioxidant enzymes (SOD, CAT), while also protecting phospholipid bilayers under stress, forming a “structural protection–enzymatic defense” dual mechanism in conjunction with the upregulation of *HSP20*. Polyamine accumulation enhanced osmotic regulation and maintained cell survival under ionic stress.

Network analysis revealed the participation of glutathione−related enzymes (**Figure 4C**). It is worth noting that *glutathione reductase* (*GR*) was strongly upregulated at 150 mM, which regenerates the GSH pool from GSSG and significantly increased the GSH/GSSG ratio.

This metabolic shift was accompanied by increased levels of downstream products including glutathione, glycine, and L−cysteine (**Figure 4D**). Functionally, GSH served as a key ROS scavenger, redox regulator, heavy metal detoxifier, and protector of macromolecular stability. Glycine contributed to osmotic adjustment and stress−protein synthesis, while L−cysteine acted both as an antioxidant and a precursor for glutathione biosynthesis.

### 3.9 Results of qRT-PCR validation of key stress resistance genes

**Figure 5:**
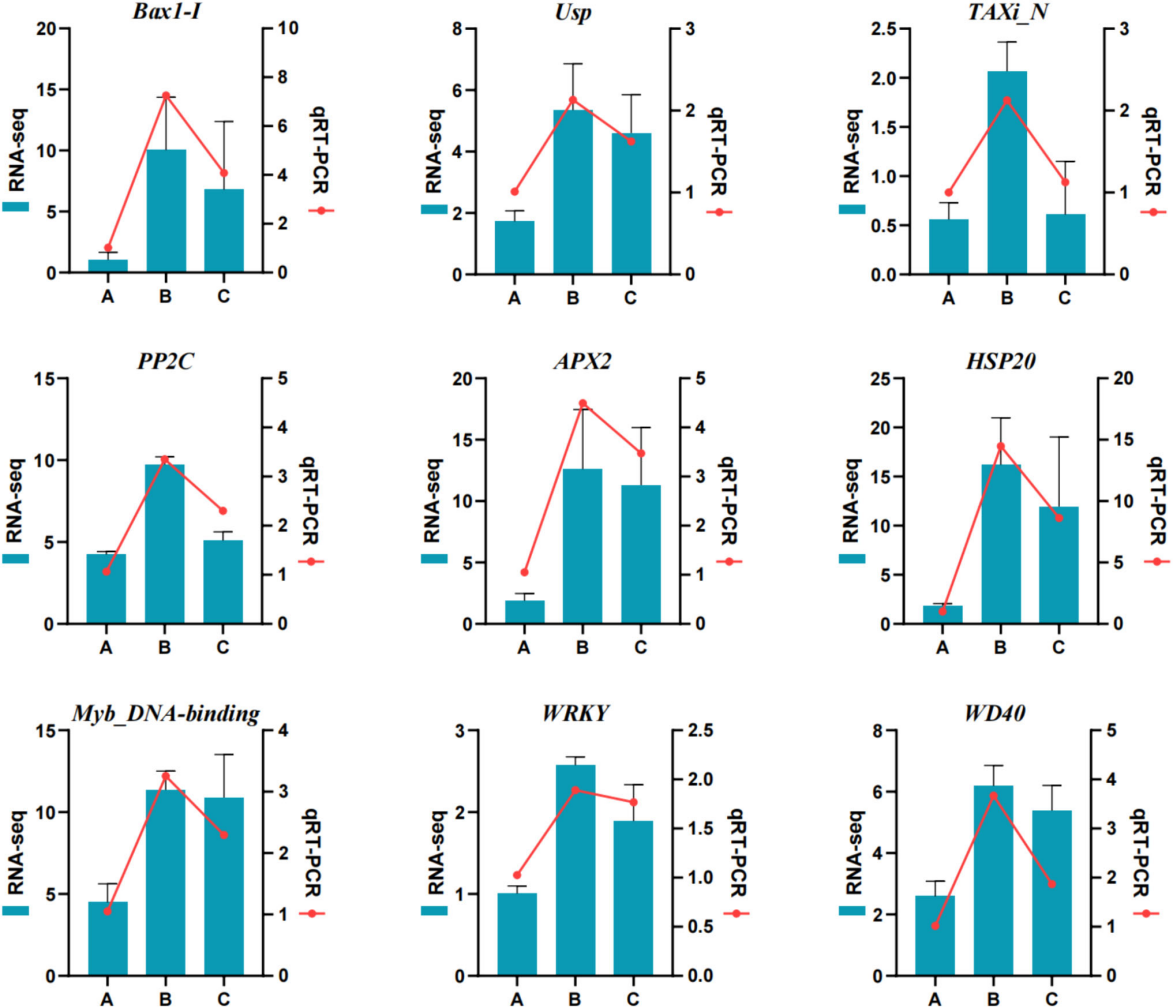
qRT-PCR validation of nine representative stress-related genes under different calcium concentrations (0 mM, 30 mM, and 150 mM). Blue bars represent normalized expression levels obtained from RNA-seq analysis, while red lines and markers indicate relative expression levels determined by qRT-PCR. Consistent expression trends between RNA-seq and qRT-PCR analyses confirm the reliability of the transcriptomic data.

To validate the transcriptomic data, nine stress-related genes were selected for qRT-PCR analysis, including antioxidant genes (*PP2C, APX2, HSP20*), transcription factors (*WRKY, MYB*), and other stress-associated genes (*Bax1-I, Usp, TAXi_N, WD40*). The expression profiles obtained by qRT-PCR (red lines) were highly consistent with RNA-seq results (blue bars), confirming the reliability of the transcriptomic dataset (**Figure 5**). The expression of *APX2* and *HSP20* was significantly induced under calcium stress, with *APX2* upregulation supporting the activation of antioxidant defense mechanisms. The transcription factors *WRKY* and *MYB* were significantly induced in a calcium-responsive manner. The relative expression of the *MYB* transcription factor showed 2.5-fold and 2.2-fold upregulation under 30 mM and 150 mM calcium stress, respectively. It is worth noting that most genes reached peak expression levels under moderate calcium stress (30 mM) and exhibited relatively lower induction under severe stress (150 mM). This suggests that *P. aizoon* optimizes the induction of stress-responsive genes under moderate stress (30 mM) to enhance tolerance while conserving energy. Under severe stress (150 mM), a potential shift in regulatory strategies may occur to prevent metabolic exhaustion and minimize cellular damage when homeostasis is severely challenged.

### 3.10 Gradient calcium stress synergistically induces flavonoid synthesis and glutathione metabolism pathway activation

**Figure 6:**
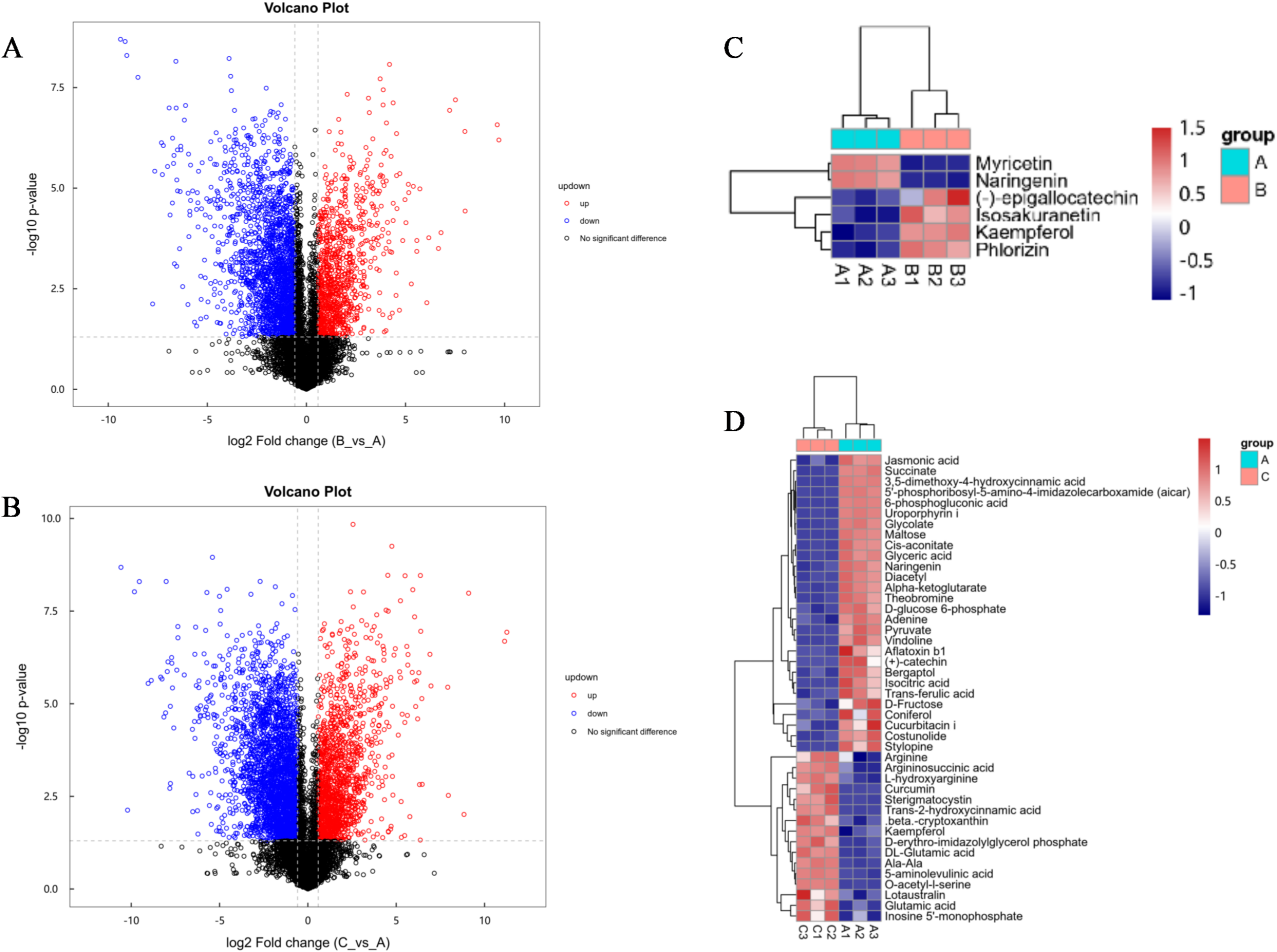
Statistics and clustering analysis of differentially expressed metabolites (DEMs) under calcium stress. **(A, B)** Volcano plots of DEMs in **(A)** Group B (30 mM CaCl_2_) and **(B)** Group C (150 mM CaCl_2_) compared to the control. **(C, D)** Cluster heatmaps of DEMs in **(C)** Group B and **(D)** Group C.

Volcano plot analysis showed that calcium stress significantly altered the metabolic reprogramming pattern in P. aizoon (**Figures 6A–D**). When treated with 30 mM CaCl_2_ (Group B), several flavonoids, including EGC, isoquercitrin, kaempferol, and gallocatechin, showed significant upregulation with log_2_ fold change > 0.5 (**Figure 6A**). This pattern indicates that moderate calcium stress preferentially stimulates flavonoid biosynthesis as an early metabolic response. When severe stress was applied (150 mM CaCl_2_, Group C), the range of metabolites showing extreme variation (|log_2_FC| ≥ 1) was markedly expanded to include glutathione (GSH), flavonoid glycosides, and amino acid derivatives (**Figure 6B**). Compared with moderate stress, severe calcium exposure induced a broader and more intensive metabolic remodeling, suggesting a dose-dependent amplification of defense-related metabolism.

Heatmap clustering analysis revealed distinct functional modules of regulated metabolites (**Figures 6C, D**). In the flavonoid–organic acid module of Group B, the mean correlation coefficient among metabolites was 0.68, and citric acid showed a negative correlation with calcium ions (**Figure 6C**). This module-level coordination suggests a functional coupling between organic acid metabolism and flavonoid accumulation under moderate calcium stress. Interestingly, kaempferol showed a 1.51-fold increase compared with the control (Group A), implying a potential role in calcium chelation and antioxidant defense. In contrast, Group C displayed a carbohydrate-associated module in which metabolites such as glucose were strongly downregulated, while an antioxidant-enriched module (GSH and flavonoids) was significantly activated, with an average upregulation of 1.28-fold relative to the control. It is worth noting that kaempferol was consistently upregulated under both 30 mM and 150 mM treatments, identifying it as a core defense-associated metabolite across calcium stress intensities.

### 3.11 Distribution characteristics and cluster analysis of differential metabolites

**Figure 7:**
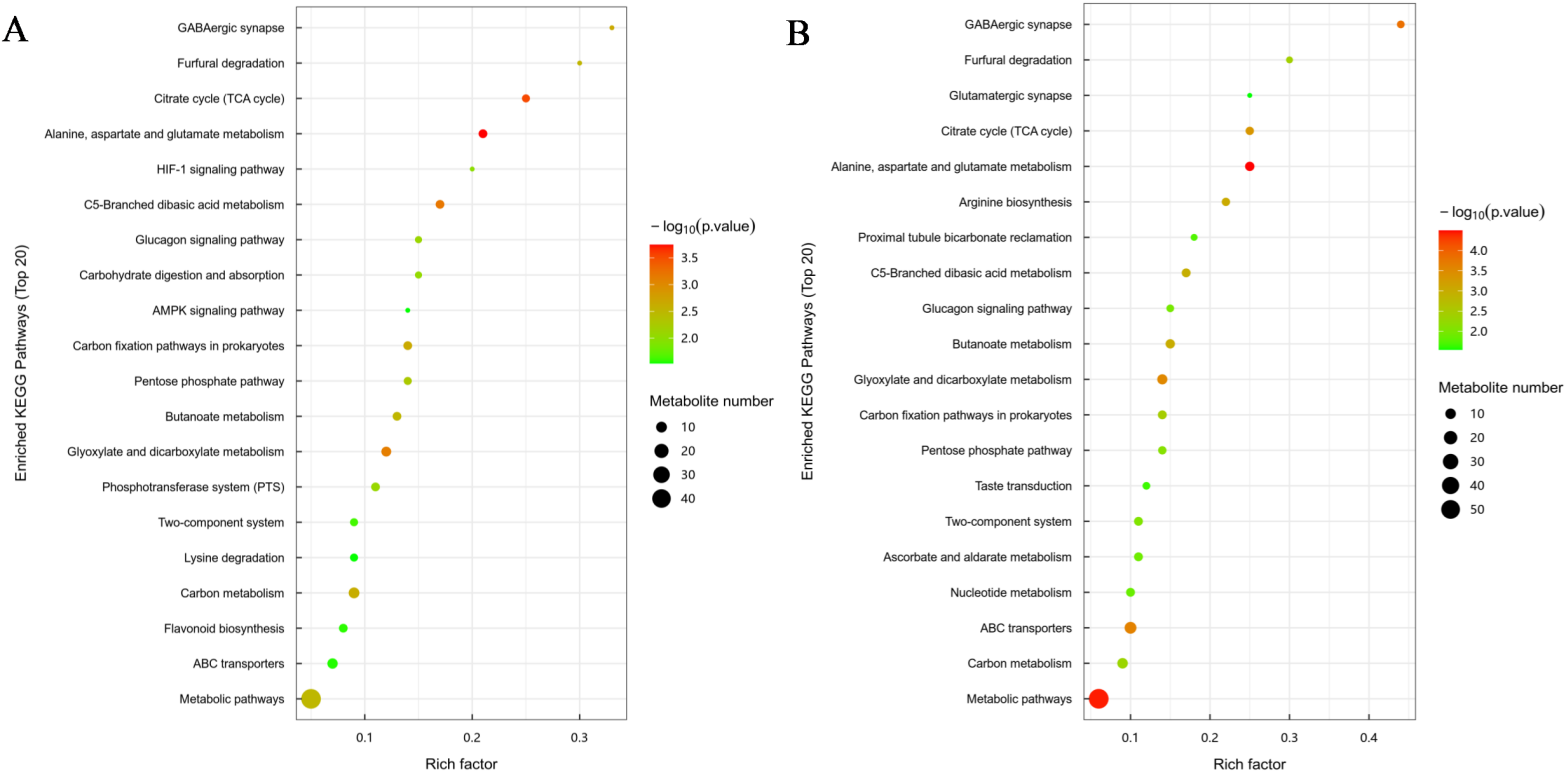
KEGG pathway enrichment analysis of differentially expressed metabolites (DEMs) in *P. aizoon* under **(A)** moderate calcium stress (30 mM CaCl_2_, Group B) and **(B)** severe calcium stress (150 mM CaCl_2_, Group C). The x-axis represents the Rich factor, circle size indicates the number of metabolites, and circle color represents the significance level expressed as −log10(p.value)−log10​(p.value).

Under moderate calcium stress (30 mM CaCl₂), pathway enrichment analysis showed that the GABAergic synapse pathway exhibited the highest Rich factor (0.33) (**Figure 7A**). Gamma-aminobutyric acid (GABA), a key regulator of stress adaptation, is known to modulate ROS metabolism, stabilize signaling cascades, and enhance antioxidant enzyme activity. The enrichment of GABA-related pathways suggests that moderate calcium stress primarily activates metabolic routes associated with stress signaling and redox regulation rather than extensive metabolic disruption. The flavonoid biosynthesis pathway was also significantly enriched (Rich factor = 0.081, p < 0.05), encompassing 6 flavonoid metabolites, including myricetin (log₂FC = 2.3) and hesperidin (log₂FC = 1.8). In addition, enrichment of the TCA cycle and alanine–aspartate–glutamate metabolism indicated accumulation of citric acid and glutamic acid, which may contribute to Ca²⁺ chelation and provide carbon skeletons for secondary metabolite biosynthesis. These coordinated enrichments imply that moderate calcium stress promotes metabolic flexibility while maintaining primary metabolic balance.

In contrast, exposure to severe calcium stress (150 mM CaCl₂) triggered a more complex and intensified defense network (**Figure 7B**). Alanine, aspartate and glutamate metabolism was markedly enriched (Rich factor = 0.25), which likely provided crucial precursors, such as glutamic acid, for glutathione (GSH) biosynthesis—a core defense mechanism against severe oxidative stress. Upregulation of metabolic flux toward maintaining a high redox buffering capacity was further evidenced by the significant enrichment of the GABAergic synapse pathway (Rich factor = 0.44). Meanwhile, secondary metabolite pathways were further activated, consistent with the induction of stress-responsive transcription factors (**Figure 7B**). Additionally, detoxification and transport processes were reinforced, as evidenced by significant enrichment of the ABC transporter pathway (Rich factor = 0.10) (**Figure 7B**). These results suggest that severe calcium stress engages both metabolic and transport-based defense mechanisms.

### 3.12 Differential abundance score analysis of metabolic pathways

**Figure 8:**
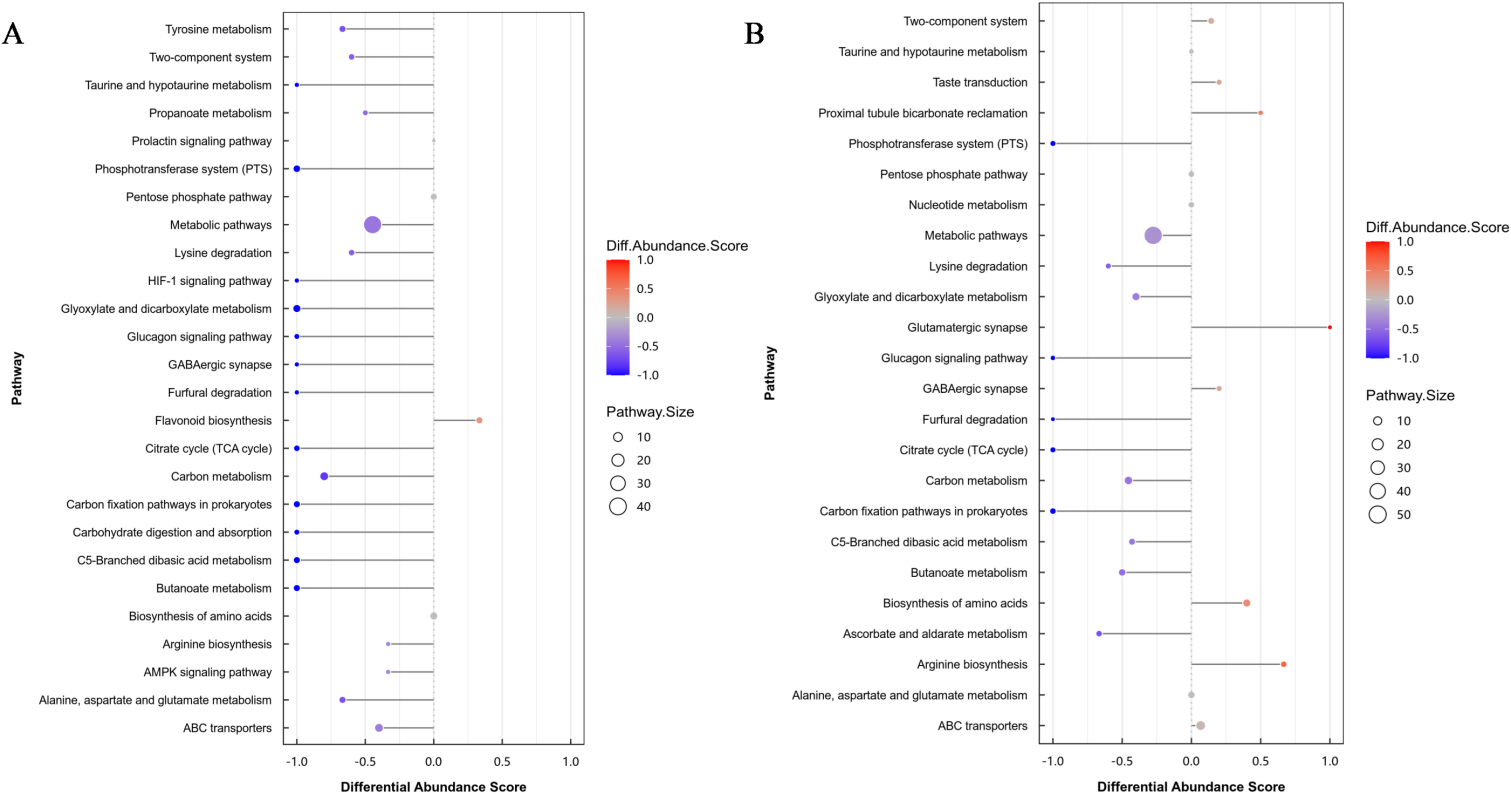
Differential abundance (DA) score analysis of metabolic pathways in Phedimus aizoon under **(A)** moderate (30 mM CaCl_2_) and **(B)** severe (150 mM CaCl_2_) calcium stress. Positive DA scores indicate pathway enrichment, whereas negative values indicate pathway suppression.

Differential abundance (DA) score analysis revealed distinct pathway reprogramming patterns under moderate (30 mM) and severe (150 mM) calcium stress (**Figure 8**). Under 30 mM CaCl_2_ treatment (**Figure 8A**), flavonoid biosynthesis exhibited the highest positive DA score (> 0.25), indicating its dominant role in secondary metabolic adjustment. Glutathione metabolism and the TCA cycle were also moderately enhanced (DA score > 0.25), reflecting their involvement in ROS scavenging and metabolic flux redistribution. In contrast, slight downregulation of carbon metabolism (DA score < −0.1) suggests a limited reallocation of resources toward defense processes under moderate stress.

When CaCl_2_ concentration reached 150 mM (**Figure 8B**), stress responses were further intensified. Glutathione metabolism displayed higher positive DA scores compared with 30 mM treatment, while the ABC transporter pathway was strongly activated, indicating enhanced sequestration and detoxification capacity. At the same time, persistent suppression of carbon and energy metabolism pathways (DA score < −0.5) suggests a strategic shift from growth-related metabolism toward survival-oriented defense under severe calcium stress.

## 4 Discussion

Calcium is an essential macronutrient that contributes to cell wall stabilization, membrane integrity, and regulation of diverse physiological processes in plants (Dodd et al., 2010; White and Broadley, 2003; Feng et al., 2023). As a universal second messenger, Ca²^+^ plays a central role in translating environmental and developmental cues into cellular responses. Against this background, the enhancement of defense systems observed in this study may be linked to Ca²^+^-mediated signaling processes that coordinate antioxidant machinery and secondary metabolism. At the same time, while adequate calcium supply is required for normal growth and development, excessive calcium accumulation can disturb cellular homeostasis and induce stress responses (Yan et al., 2024). *P. aizoon* is a vegetable species with both nutritional and medicinal value, characterized by a naturally high calcium content together with abundant bioactive metabolites such as flavonoids and ginsenosides (Gao et al., 2024). Our previous study (Zhang and Luo, 2026) demonstrated that *P. aizoon* exhibits a significant 1.56−fold (~56%) increase in stem soluble calcium under karst conditions, supporting its suitability as a non−model system for investigating plant responses to calcium gradients.

### 4.1 Growth and physiological responses to calcium availability

Our results indicate that *P. aizoon* exhibits a clear concentration−dependent response to Ca²^+^ availability. Moderate Ca²^+^ supply (30 mM CaCl_2_) promoted vegetative growth, as reflected by increased plant height and crown breadth, whereas excessive Ca²^+^ (150 mM CaCl_2_) resulted in growth inhibition and chlorosis. This moderate−promotion and severe−suppression pattern is consistent with classical observations that calcium supports growth within an optimal range but becomes restrictive when accumulated beyond the buffering capacity of cellular homeostasis (White and Broadley, 2003; Feng et al., 2023).

Physiological measurements further showed that moderate Ca²^+^ supply was associated with enhanced antioxidant enzyme activities, including SOD and CAT, together with increased accumulation of soluble sugars, proteins, and phenolic compounds. By contrast, under severe Ca²^+^ stress, growth was suppressed even though lipid peroxidation did not increase markedly, suggesting that core antioxidant defenses remained partially operative despite reduced biomass accumulation. Such growth–defense trade−offs are widely observed under abiotic stress conditions, reflecting a shift in resource allocation toward cellular protection (Blumwald, 2000; Munns and Tester, 2008).

### 4.2 Transcriptomic reprogramming under calcium stress

Transcriptomic analyses revealed broad gene expression reprogramming in response to calcium stress, indicating that Ca²^+^ availability elicits systemic transcriptional adjustments rather than isolated gene−level responses. Under moderate Ca²^+^ treatment (30 mM), differentially expressed genes were predominantly enriched in GO categories related to stress response, redox regulation, and secondary metabolism, whereas severe Ca²^+^ stress (150 mM) triggered a wider transcriptional response that was less supportive of growth. KEGG enrichment further highlighted phenylpropanoid biosynthesis and glutathione metabolism as major calcium−responsive pathways, consistent with the established role of calcium in stress signaling and redox regulation (Dodd et al., 2010; Ahmed et al., 2021).

qRT−PCR validation of nine representative stress−related genes confirmed the reliability of the RNA−seq data and provided molecular support for calcium−induced stress responses. Under moderate calcium stress, *APX2* expression increased by approximately 3.5−fold, suggesting enhanced antioxidant capacity. This response is consistent with previous studies showing that Ca²^+^−induced ROS production activates ascorbate peroxidase−mediated ROS scavenging under calcium or salt stress (Mittler et al., 2004; Foyer and Noctor, 2005). Similarly, *HSP20* showed an approximately four−fold induction, indicating activation of protein quality control mechanisms that protect cellular proteostasis under ionic and oxidative stress (Wang et al., 2004; Hua et al., 2023).

In addition, the transcription factors *WRKY* and *MYB* were significantly induced, highlighting transcriptional reprogramming downstream of calcium signaling. Both transcription factor families are known to function as key integrators of Ca²^+^ and ROS signals, coordinating stress−responsive integrators under calcium and salt stress conditions (Rushton et al., 2010; Dubos et al., 2010). It is worth noting that most of these genes reached peak expression under moderate calcium stress but declined under severe stress, suggesting a biphasic response pattern. This “low−calcium promotion and high−calcium inhibition” response implies that *P. aizoon* fine−tunes stress−resistance mechanisms to balance protection and energy expenditure under varying calcium conditions. Consistent with this interpretation, genome−wide studies have shown that calcium−signaling components function as central integrators of abiotic stress responses in plants (Narwal et al., 2024b).

### 4.3 Metabolic remodeling in response to calcium gradients

Metabolomic profiling indicated that metabolic responses to calcium stress followed a graded pattern across treatments rather than irregular or stochastic fluctuations. Moderate calcium stress preferentially enhanced flavonoid biosynthesis and stress−related signaling metabolites, whereas severe calcium exposure triggered broader metabolic reprogramming involving glutathione metabolism, amino acid derivatives, and suppression of primary carbon metabolism. This dose−dependent metabolic pattern is consistent with transcriptomic results showing increasingly pronounced transcriptional reprogramming with rising calcium levels. Similar graded metabolic responses to abiotic stress have been reported in plants as a strategy to balance growth and defense investment (Dixon and Paiva, 1995; Hasanuzzaman et al., 2020; Ahmed et al., 2021).

One notable metabolic response to calcium stress was the simultaneous activation of flavonoid biosynthesis and glutathione metabolism. Flavonoids such as kaempferol, hesperidin, and epigallocatechin accumulated under both moderate and severe stress, providing antioxidant capacity and potential calcium−chelating functions (Pietta, 2000). In parallel, enhanced glutathione metabolism under severe calcium stress, together with increased availability of its precursors, suggests reinforcement of cellular redox buffering. Together, these responses indicate that antioxidant protection under calcium stress relies on multiple, interconnected metabolic components rather than a single pathway. Such coordination is consistent with transcriptional induction of *MYB* and *WRKY* transcription factors, which are known regulators of flavonoid biosynthesis and stress−responsive antioxidant pathways (Szabados and Savouré, 2010; Hasanuzzaman et al., 2020).

Under severe calcium stress, metabolic adjustments were accompanied by enhanced transport−related processes, particularly the activation of ABC transporters. This suggests that intracellular compartmentalization of flavonoids, glutathione−related compounds, and calcium−associated complexes may reduce cytosolic ion toxicity and contribute to redox homeostasis. In parallel with the suppression of carbon and energy metabolism, these changes indicate a reallocation of metabolic resources from growth−associated processes toward stress defense. Similar calcium−mediated enhancement of antioxidant capacity, osmotic regulation, and secondary metabolite accumulation has been reported in other stress−resilient species (Narwal et al., 2024a; Narwal and Negi, 2024). Collectively, these metabolic features provide a conceptual basis for the transcriptional–metabolic coordination model proposed below.

### 4.4 A proposed transcriptional–metabolic coordination model underlying calcium stress adaptation

**Figure 9:**
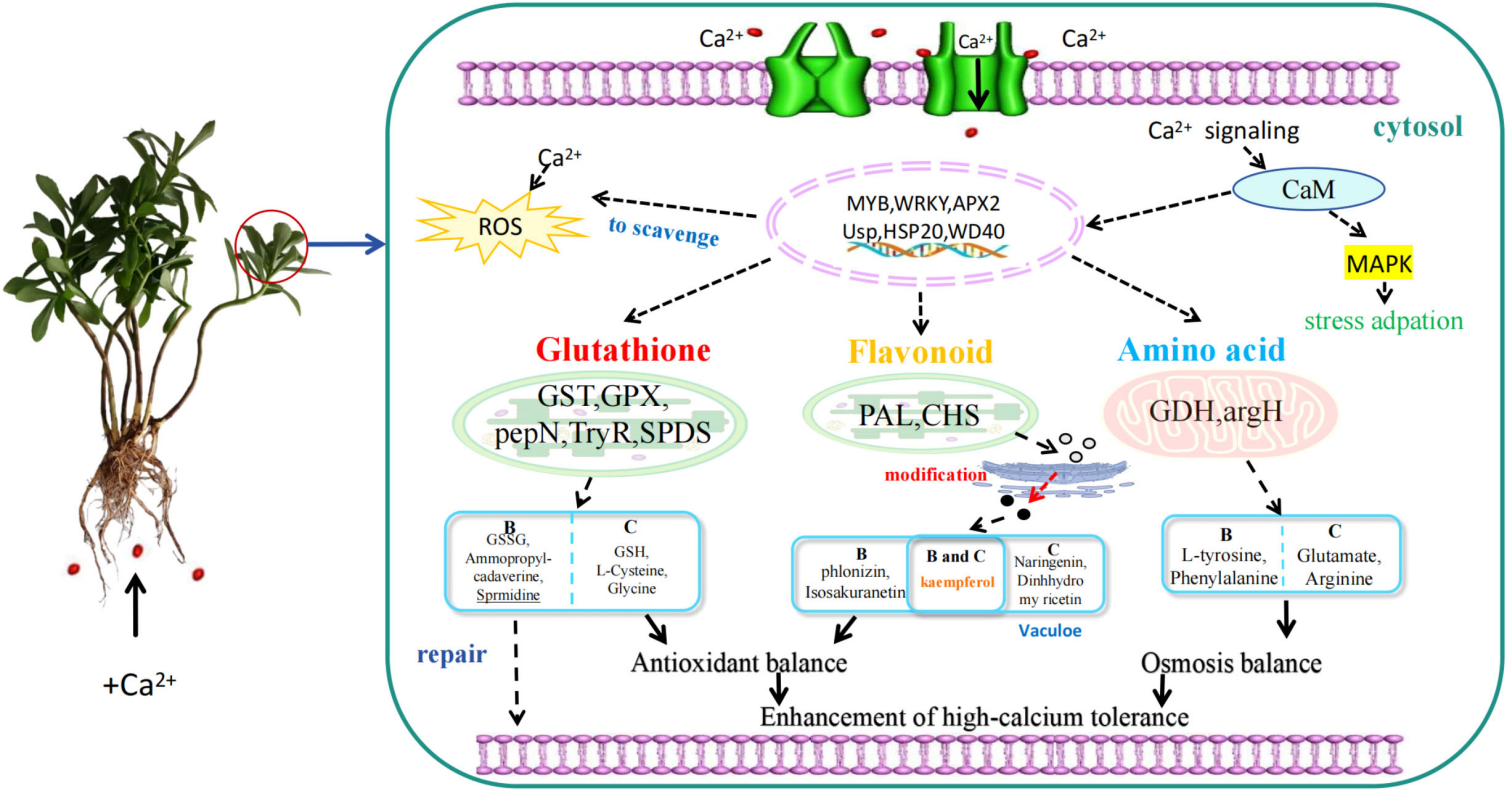
Conceptual transcriptional–metabolic coordination model underlying calcium stress adaptation in *Phedimus aizoon*. The model summarizes coordinated transcriptional and metabolic responses to increasing calcium availability inferred from integrated transcriptomic and metabolomic analyses. Calcium stress is associated with induction of WRKY and MYB transcription factors and coordinated activation of flavonoid biosynthesis, glutathione metabolism, and amino acid metabolism, contributing to redox homeostasis and ionic balance. Enhanced transporter activity under severe calcium stress may facilitate intracellular compartmentalization of secondary metabolites and calcium-associated complexes. This schematic representation is correlative and does not imply a definitive molecular mechanism.

Based on integrated transcriptomic and metabolomic analyses across a gradient of calcium treatments, we propose a conceptual transcriptional–metabolic coordination model to summarize potential regulatory relationships underlying calcium stress adaptation in *P. aizoon* (**Figure 9**). This model reflects correlative multi−omics patterns rather than a definitive molecular mechanism. At the transcriptional level, calcium stress was associated with the induction of stress−responsive transcription factors, particularly members of the *WRKY* and *MYB* families, which are widely implicated in the regulation of antioxidant defense and secondary metabolism. The coordinated activation of these regulators provides a plausible framework linking calcium−responsive transcriptional reprogramming to downstream metabolic adjustments in redox−related and stress−responsive pathways.

Consistent with this framework, metabolomic analyses revealed coordinated activation of multiple antioxidant−related metabolic pathways. Enhanced phenylpropanoid–flavonoid metabolism, together with plasticity in amino acid metabolism and reinforcement of glutathione−based redox buffering, suggests that calcium stress elicits an integrated antioxidant response rather than reliance on a single detoxification mechanism. In addition, transport−related processes, including ABC transporter activity, may facilitate intracellular compartmentalization of secondary metabolites and calcium−associated complexes, thereby alleviating cytosolic ion stress.

It is worth noting that the extent of transcriptional and metabolic adjustment depended on calcium intensity. Moderate calcium supply was associated with balanced activation of antioxidant metabolism, consistent with maintenance of growth and basal stress protection, whereas severe calcium stress coincided with intensified defense−related metabolism, extensive amino acid reprogramming, and growth suppression, reflecting a trade−off between growth and stress defense. Similar response patterns have been observed in other stress−resilient species exposed to elevated calcium conditions (Narwal et al., 2024a; Narwal and Negi, 2024). While supported by integrative multi−omics evidence, this model remains correlative and will require targeted functional validation to clarify the underlying regulatory mechanisms.

### 4.5 Ecological and agronomic implications

The physiological plasticity observed in *P. aizoon* suggests that this species is able to adjust both growth and antioxidant metabolism across a range of external calcium conditions, which may help explain its occurrence in calcium-rich habitats. This pattern is consistent with observations in other plant systems, where calcium is linked to broader metabolic and signaling networks involved in stress adaptation and secondary metabolism (Li et al., 2023; Guo et al., 2024). In our study, moderate and high CaCl_2_ treatments resulted in clearly different outcomes. Moderate concentrations were associated with better growth and antioxidant capacity, whereas high concentrations inhibited growth and were accompanied by sustained defense-related activity. These findings are relevant to the cultivation of *P. aizoon*, particularly in relation to its edible and medicinal use rather than ornamental performance. From a production perspective, the growth inhibition observed at high treatment levels would reduce its cultivation value. However, moderate calcium treatment may still be worthwhile if it improves the nutritional or medicinal quality of the edible shoots.

In natural karst soils, calcium occurs mainly as CaCO_3_, and plant access to Ca^2+^ is governed by gradual mineral dissolution. The soilless system used here provided a controlled way to examine plant responses to soluble calcium. While CaCl_2_ introduces chloride ions, it has been demonstrated to be a more effective inducer of ROS accumulation and flavonoid biosynthesis compared to other calcium salts like Ca(NO_3_)_2,_ which may instead exert a mitigating effect due to its nitrogen content (Liu et al., 2025). Therefore, our results should be interpreted as specific responses to CaCl_2_-mediated stress under controlled conditions. While CaCl_2_ was the primary source tested to isolate the calcium signaling and stress response without nitrogen interference, future comparisons with CaCO_3_ and Ca(NO_3_)_2_ under diverse cultivation conditions will further validate these findings.

## 5 Conclusion

The response of *P. aizoon* to CaCl_2_ treatment varied with concentration. Moderate treatment was associated with better growth, whereas excessive treatment led to growth inhibition and stronger stress-related responses. Integrated transcriptomic and metabolomic analyses suggested that flavonoid- and glutathione-related pathways may be involved in maintaining redox balance under high-calcium treatment. Overall, this study provides a useful basis for further work on calcium-related responses in P. aizoon and offers preliminary insight into the potential effects of calcium supply on its nutritional and medicinal quality.

## Data Availability

The datasets presented in this study can be found in online repositories. The names of the repository/repositories and accession number(s) can be found below: https://ngdc.cncb.ac.cn/gsa., (GSA: CRA032169). Metabolite qualitative and quantitative data are provided as **Supplementary Data 1**.
